# Sheng-Jiang Powder Ameliorates High Fat Diet Induced Nonalcoholic Fatty Liver Disease via Inhibiting Activation of Akt/mTOR/S6 Pathway in Rats

**DOI:** 10.1155/2018/6190254

**Published:** 2018-10-04

**Authors:** Juan Li, Lv Zhu, Yu-mei Zhang, Huan Chen, Yi-fan Miao, Hong-xin Kang, Hong-yu Ren, Mei-hua Wan, Dan Long, Wen-fu Tang

**Affiliations:** ^1^Department of Integrated Traditional Chinese and Western Medicine, West China Hospital, Sichuan University, Chengdu 610041, Sichuan Province, China; ^2^Key Laboratory of Transplant Engineering and Immunology, Sichuan University, Chengdu 610041, Sichuan Province, China

## Abstract

**Background and Aims:**

Nonalcoholic fatty liver disease (NAFLD) is an alarming public health problem that directly contributes to increased prevalence of liver cirrhosis and hepatic cell cancer, but without any specific pharmacological option. Sheng-jiang powder (SJP), an empirical Chinese medicine formula to treat NAFLD, showed great efficacy but the specific mechanisms have never been reported. Therefore, we performed this study to explore the effect of SJP on NAFLD and the potential mechanism.

**Methods:**

NAFLD was induced by high fat diet (HFD) feeding. Rats were treated with SJP/normal saline daily for 10 weeks and all rats were euthanized after 12 weeks' feeding. Liver tissue samples were obtained for biochemistry test and pathological evaluation. Additionally, oleic acid induced LO2 cells were employed to simulate a cell model of NAFLD. Cells were subjected to western blotting for Akt, mTOR, S6, SREBP1-c, and FASN detection after coincubated with SJP, LY294002 (a selective PI3K inhibitor), or the combination for 24h.

**Results:**

HFD significantly induced hepatic steatosis. Plenty of lipid droplets were observed under transmission electron microscope. The ultrastructure of liver cells showed distinct changes with obvious endoplasmic reticulum expansion, mitochondrial contraction, and cell matrix solidification. Although no difference was detected in serum hepatic enzymes and tissue proinflammatory cytokines, the tissue level of SOD and GSH-px was much lower and the pathologic injuries were much severe in HFD feeding rats. However, SJP treatment significantly attenuated the ultrastructure changes and protected rat liver against inflammatory injury. Abundant of lipid droplets and high expression of pAkt, pmTOR, pS6, and FASN were observed in oleic acid treated LO2 cells, while these changes were restored by SJP treatment.

**Conclusions:**

SJP is efficient in attenuating HFD induced NAFLD in rats and this effect might be partly related to the inhibition of Akt/mTOR/S6 pathway.

## 1. Introduction

Nonalcoholic fatty liver disease (NAFLD) is an alarming public health problem that is directly linked to the epidemic of obesity and contributes to increased prevalence of liver cirrhosis and hepatic cell cancer [1, 2]. As the most common chronic liver disease, NAFLD affects one-quarter of the population around the word and leads to enormous clinical and economic burden [3].

NAFLD is characterized by excessive fat accumulation in hepatocytes and ranges from simple hepatic steatosis to nonalcoholic steatohepatitis (NASH), hepatic fibrosis, and finally cirrhosis [4, 5]. It is highly associated with several kinds of metabolic disorders, such as hyperlipidemia and insulin resistance [6, 7]. In fact, a clear route of “insulin resistance-abnormal lipid metabolism- NAFLD” has become a consensus which threw a light on the mechanism and treatment research on NAFLD [8, 9]. PI3K/ Akt signaling pathway is the main downstream pathway of insulin [10]. Activated Akt by the phosphorylation of Thr308 and Ser473 sites initiates the downstream substrate protein cascade reaction and then participates in glycogen synthesis, glucose transporter, glycolytic protein synthesis, and glycogenetic inhibition as well as lipid synthesis and decomposition which are crucial to energy hemeostasis [11, 12]. Therefore, dysregulated Akt activity may contribute to a series of disorders in human body including NAFLD [11, 13]. However, the role of Akt in the development of NAFLD is still controversial because of paradoxical results showed by different studies. Briefly, some studies found an obvious activation of sterol regulatory element binding protein 1c (SREBP-1c) followed by Akt activaiton, which promoted fat deposition in liver [14], while other studies revealed a distinct opposite role of Akt activation for protecting against hepatic steatosis [15, 16]. Although no consistent view was achieved on this issue, it did indicate an important role of Akt in the pathogenesis of NAFLD at least.

Currently, the main therapeutic strategies for NAFLD depend on lifestyle changes as there is no specific pharmacological option for the treatment of NAFLD [17–19]. But due to poor adherence to life style modification, finding new therapeutic agents to treat NAFLD or preventing its progression has attracted many interests. Statins and fibrates may help lower incidence of NAFLD in a small portion of cases via reducing blood cholesterol and triglycerides levels [20], there is still an urgent need to develop a new efficient approach for the management of NAFLD for majority of patients and now Chinese herbal medicine has garnered significant interest. According to the theory of traditional Chinese medicine, NAFLD belongs to the category of “Liver turbidity”. An unhealthy diet with high fat and calories intake causes damage to the transport function of spleen which plays a central role in the motion of qi. Normal transportation and spreads of nutrients depend on normal motion of spleen qi. Thus, abnormal motion of spleen qi causes qi stagnation, phlegm retention, and further blood stasis which block the liver collaterals and finally induces the development of NAFLD [21]. SJP, derived from “wan bing hui chun” which was compiled by ting-xian gong during the Ming dynasty of China, was a classic representative Chinese herbal medicine formula to restore “abnormal ascending and descending function” of spleen qi and was empirically applied in the treatment of NAFLD for decades [22]. Researches have demonstrated a wide range of effects for SJP, such as losing weight, anti-inflammation, antiviral, antiallergic, and antipyretic analgesia as well as immune regulation [23]. Some Chinese studies have showed remarkable efficacy of SJP on NAFLD, insulin resistance as well as metabolic syndrome [24, 25]. Additionally, our previous study has demonstrated an effective role of SJP in lowering body weight and attenuating liver injury in obese rats [26]. However, there has been no study focusing on the mechanism of SJP in protecting against NAFLD so far. Thus, given the impressive effect observed by Chinese researchers from long-term practices, substantial interest was developed to explore the effect of SJP on NAFLD and the potential mechanism, especially the PI3K/Akt signal pathway.

## 2. Materials and Methods

### 2.1. Design

Prospective, randomized controlled trial.

### 2.2. Settings

Key laboratory of transplant engineering and immunology, Sichuan University.

### 2.3. Ethics Statement

The protocol was approved by the Ethics Committee for Animal Experiments of Sichuan University. All rats were handled according to the University Guidelines and the Animal Care Committee Guidelines of West China Hospital. All surgeries were performed under pentobarbital anesthesia, and all efforts were made to minimize suffering of rats.

### 2.4. Preparation of Sheng-Jiang Powder

SJP was derived from the famous Chinese medical book “wan bing hui chun” and was composed by Jiangchan (*Bombyx Batryticatus*, 6g), Chantui (*periostracum cicada*, 3g), Jianghuang (*Curcuma longa*, 9g), and Dahuang (raw rhubarb, 12g). The spray-dried Jiangchan (1701117), Chantui (1608027), Jianghuang (1506067), and Dahuang (1610039) powder were purchased from Chengdu New Green Herbal Pharmaceutical Co., Ltd. (Chengdu, China). The spray-dried powder was mixed according to the original compatibility proportion of the crude drugs and reconstituted with distilled water at a concentration for the crude drug of 1g/ml SJP. According to the original prescription recorded, the dose of an adult was 0.5g/Kg.BW. Therefore, we adopt a 10-fold dose (5g/Kg.BW) to treat the experimental animals. In vitro study, the mixed powder was reconstituted with PBS to prepare a 0.01g/ml solution to ensure a final content of rhein 5ug/ml according to the maximum concentration we detected in the serum of rats and the mean content of rhein in SJP analyzed by HPLC [27, 28]. Solution was filtered before adding to the cells.

### 2.5. Animals and Treatment

**Figure 1:**
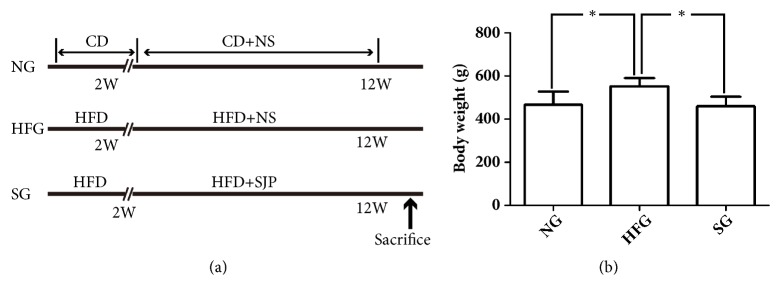
Study design of feeding methods and the body weight of rats with high fat diet feeding with/without Sheng-jiang powder (SJP) administration. Normal group (NG), high fat diet feeding group (HFG), and SJP treatment group (SG). CD: control diet; HFD: high fat diet; NS: normal saline; SJP: Sheng-jiang powder. (a) Feeding and intervention methods of the study. (b) Body weight of rats before execution. The whole study lasted for 12 weeks with 10 week's administration of SJP (5g/Kg) one time a day. All rats were sacrificed after 12 weeks' feeding.

Male Sprague-Dawley rats weighted 60-80g were purchased from Chengdu Dashuo Experimental Animal Co., Ltd. (Chengdu, China). All animals were kept under controlled temperature (22-23°C) and on a 12-h light/12-h dark cycle and followed free access to a HFD (60% of calories derived from fat; TP23300; Trophic Animal Feed High-tech Co., Ltd., China) to induce NAFLD or control diet (16.7% of calories derived from fat; TP23302; Trophic Animal Feed High-tech Co., Ltd., China) (http://trophic.biomart.cn). Animals were randomly allocated to normal group (NG, control diet, n=6), high fat diet group (HFG, high fat diet, n=8), and SJP treatment group (SG, high fat diet plus SJP, n=8) by random number table. The whole study lasted for 12 weeks with 10-week administration of SJP (5g/Kg) one time a day and body weight was recorded every week. Rats in SG were gavaged with SJP from the third week, while rats in another two groups were gavaged with equal volume of normal saline instead. All rats were euthanized after 12-week feeding (Figure 1(a)). Tissue samples were obtained for immunohistochemistry tests and histopathological analysis. This study adhered to the ARRIVE Guidelines for reporting animal research (S1 ARRIVE Checklist).

### 2.6. Cell Lines and Treatment

Human hepatocyte LO2 was obtained from Key Laboratory of Transplant Engineering and Immunology of Sichuan University. Cells were cultured in high-glucose Dulbecco's modified Eagle's medium (Gibco, 11965-092, Grand Island, USA) supplemented with 10% fetal bovine serum (Gibco, 16000-044, Grand Island, USA) and 100 U/mL penicillin-100 mg/mL streptomycin mixture (Hyclone, SV30010, USA) at 37°C and were maintained in a humidified environment containing 5% CO_2_. LY294002 (Sigma-Aldrich, Merk KGaA, Darmstadt, Germany) was dissolved in DMSO to prepare a 10mmol/L stock solution. The stock solution was diluted with PBS to prepare the working solution (10umol/L) before adding to cells and the final DMSO concentration was less than 0.1%. All experiments were carried out 24 h after cells were seeded. To investigate the protective effects of SJP against NAFLD, LO2 cells were treated with or without SJP, LY294002, or the combination prior for 30 min and then further coincubated with oleic acid (0.5mM) for another 24 h.

### 2.7. Tissue Sampling and Cytokines Analysis

All rats were euthanized after 12 week's feeding and blood samples were obtained from heart. Liver tissues were dissected immediately and collected for cytokines and to pathological analysis. Blood samples were centrifuged at 2500 rpm for 5min and the supernatants were collected for serum biochemistry test by an automatic biochemical analyzer (HITACHI, 7170A, Japan). Tissue samples were homogenized using a tissue homogenizer (Biospec Products, Bartlesville, OK). Homogenates were incubated at 4°C for 30 minutes and then centrifuged at 1000 × g for 10 minutes. Supernatants were collected for cytokine analysis. Malondialdehyde (MDA), superoxide dismutase (SOD), glutathione peroxidase (GSH-px), and reactive oxygen species (ROS) were measured by means of enzyme-linked immunosorbent assay (ELISA) (eBio, Wuhan, China) with commercially available materials. According to the manufacturer's protocol, absorbance was measured at 450nm with High Throughput Universal Microplate Assay. The sample values were then read off the standard curve and calculated the relative concentrations.

### 2.8. Ultrastructure Detection of Liver Cells

Fresh liver tissues were cut into slices and first fixed in 3% glutaraldehyde for 24h and then fixed in 1% osmic acid for another 2h. After three times of 0.1mmol/L phosphate buffer solution (PBS) washing and different concentrations of ethanol and acetone dehydration, the little slices were embedded in epoxy resin and polymerized in 60°C incubator for 48h. Then the embedded liver tissue were sectioned into 5*μ*m slices and followed with uranium acetate-citrate staining. The ultrastructure of liver cells was observed under transmission electron microscope.

### 2.9. Oil red O Staining

Human hepatic LO2 cells were plated in 6-well plates with slides placed in advance and treated according to the experimental design described in above. Cells were washed with phosphate buffer saline (PBS) and then fixed with 4% paraformaldehyde for 30 min and stained with a freshly prepared working solution of oil red O for 1h at room temperature followed by being counterstained with hematoxylin before microscopic observation (Olympus Corporation, Tokyo, Japan). Frozen liver sections were washed with 60% isopropanol twice and then stained with freshly prepared working solution of oil red O for 1h. Then, liver sections were counterstained with hematoxylin before microscopic observation.

### 2.10. Immune Blotting of Akt/pAkt, Mtor/pmTOR, S6/pS6, SREBP1-c, and FASN

Expression of Akt/pAkt, Mtor/pmTOR, S6/pS6, SREBP1-c, and FASN were determined by western blot analysis. Briefly, LO2 cells were homogenized using a glass Dounce homogenizer in ice-cold lysis buffer (10 mM Tris/HCl, pH 7.6, 5 mM MgCl2, 1.5 mM potassium acetate, 1% Nonidet P-40, and 2 mM DTT) and 1× Halt Protease and Phosphatase Inhibitor Cocktail (Pierce, Thermo Fisher Scientific) to obtain protein lysates and protein concentrations were determined using the Bio-Rad protein assay kit (Bio-Rad Laboratories) according to the manufacturer's specifications. The lysates were then separated on an 8% SDS/PAGE. Following electrophoretic transfer on to nitrocellulose membranes and blocking with 5% milk solution, blots were incubated overnight at 4°C with primary rabbit polyclonal/monoclonal antibodies against Akt (1:1000, #9272, Cell Signaling Technology), pAkt (Ser473)(1:2000, #4060, Cell Signaling Technology), pAkt (Thr308) (1:1000, #2965, Cell Signaling Technology), mTOR(1:1000, #2972, Cell Signaling Technology), pmTOR (Ser2448)(1:1000, #5536, Cell Signaling Technology), S6 (1:1000, #2317, Cell Signaling Technology), pS6 (S240/244) (1:1000, #2215, Cell Signaling Technology), SREBP1-c (1:1000, #9874, Cell Signaling Technology), and FASN (1:1000, #3189, Cell Signaling Technology) and with a secondary antibody conjugated with horseradish peroxidase (Bio-Rad Laboratories) for 3 h at room temperature. Membranes were processed for protein detection using Super Signal substrate (Pierce) and anti-GAPDH (ABD Serotec) was used as the loading control.

### 2.11. Histopathological Analysis

Fresh tissue samples were fixed in 10% neutral formalin and embedded in paraffin and then sectioned into 5*μ*m slices and followed with hematoxylin and eosin (H&E) staining. All the histopathology specimens were reviewed and scored in a blinded fashion by two independent pathologists using a scoring system for the extent and severity of tissue injury (point 0–4, edema, neutrophil infiltration, necrosis, and hemorrhage) as previously described [29]. The total histopathology score is the mean of the combined scores for each parameter from both investigators.

## 3. Statistical Analysis

All data were expressed as mean ± SD. Statistical analysis was performed with PEMS3.1 statistical program for Windows. One-way ANOVA was used to analyze group differences in the study. Differences with a* P* < 0.05 were considered to be statistically significant.

## 4. Results

### 4.1. SJP Protected against High Fat Diet Induced Liver Steatosis in Experimental Rats

**Figure 2:**
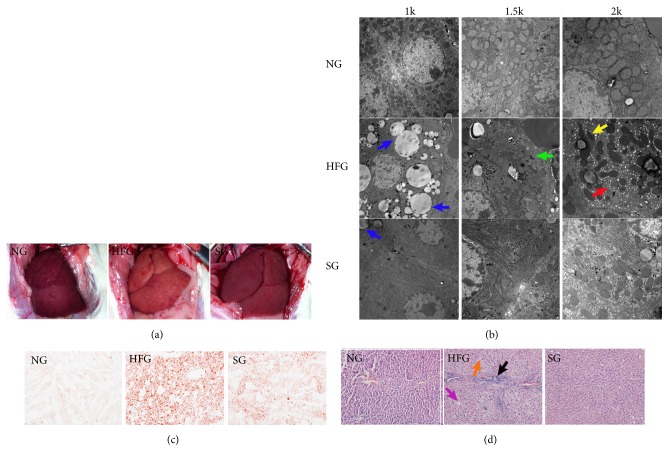
The macroscopic and microscopic appearance as well as the pathological images of liver among the three experimental groups. (a) The macroscopic appearance of liver. The fresh liver in NG showed a fine texture, dark red color and sharp edge in appearance (left), while the fatty liver appeared slightly swollen with a greasy texture, and the color turned yellow, the edge became dull in HFG (middle), and the liver tissues in SG appeared close to normal (right). (b) The microscopic appearance of liver among the three experimental groups. To better exhibit manifestation of liver in ultrastructure, we exhibited the ultrastructure images with three magnifications: 1k, 1.5k, and 2k. Plenty of lipid droplets (blue arrow) were observed by transmission electron microscope in HFG and the ultrastructure of liver cells showed apparent changes with endoplasmic reticulum expansion (red arrow), mitochondrial contraction (yellow arrow), and cell matrix solidification (green arrow). (c) Oil red O staining of frozen liver sections. The frozen liver sections showed obvious lipid droplets accumulation in HFG, while it was much better in SG. (d) Pathological images of liver. Hematoxylin-eosin counterstain. Histological images are presented with original magnification 200×. Liver in HFG exhibited enlarged hepatocytes (orange arrow), extensive vacuolization (purple arrow), and inflammatory cells infiltration (black arrow), and these changes were ameliorated in SG.

High fat diet feeding induced a significant weight gain and liver steatosis in rats. At the end of experimental period, rats in HFG appeared to be much fatter with the body weight increased by 20% more than that in NG (Figure 1(b)). However, rats in SG showed an almost similar weight gain with rats in NG. The fresh liver tissues in NG showed a fine texture, dark red color, and sharp edge in appearance, while they appeared slightly swollen with a greasy texture and the color turned yellow, the edge became dull in HFG. Additionally, plenty of lipid droplets were observed by transmission electron microscope in HFG and the ultrastructure of liver cells showed apparent changes with endoplasmic reticulum expansion, mitochondrial contraction, and cell matrix solidification. Moreover, abundant of lipid droplets were observed in frozen liver sections by oil red O staining, and the pathological images also showed cellular swelling and hepatocyte vacuolation. SJP showed significant efficacy in protecting against these changes (Figure 2).

### 4.2. SJP Ameliorated High Fat Diet Feeding Induced Liver Inflammatory Injury

High fat diet feeding induced liver steatosis and abnormal expression of inflammatory agents as well. Although no difference was detected in serum hepatic enzymes and tissue inflammatory cytokines as MDA and ROS, the tissue level of SOD and GSH-px were much lower and the pathologic injuries were much severe in high fat diet feeding rats according to our previous studies. However, SJP treatment significantly increased the tissue levels of SOD and GSH-px and protected rat liver against inflammatory injury caused by high fat diet feeding induced hepatic steatosis [26] (Table 1).

### 4.3. SJP Inhibited the Expression of pAkt, pmTOR, pS6, and FASN in Oleic Acid Stimulated Human Hepatocyte LO2 Cell Line

**Figure 3:**
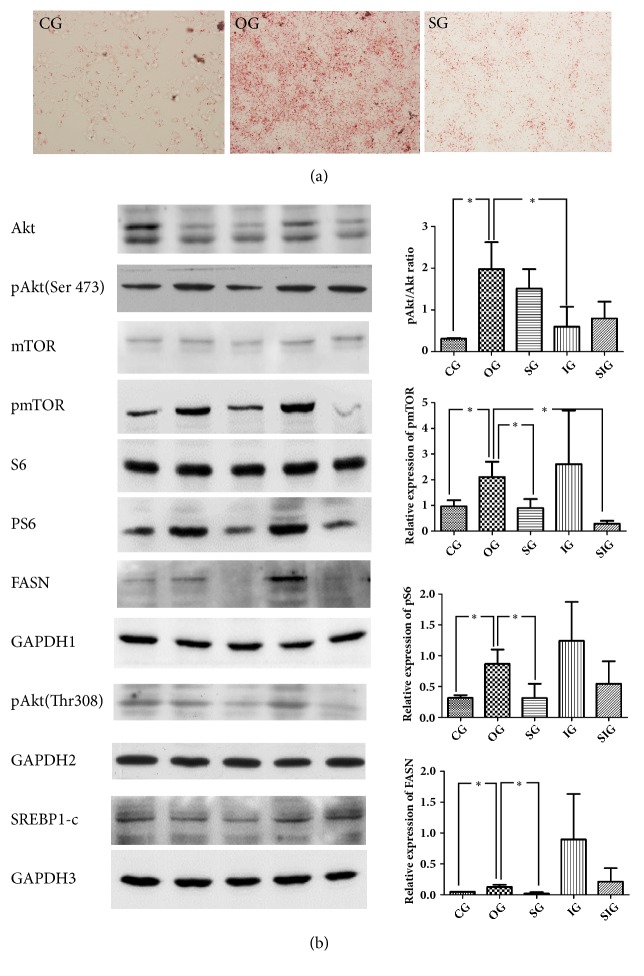
Oil red O staining and western blot of pAkt, pmTOR, and pS6. (a) Oil red O staining of LO2 cell line treated with oleic acid (0.5mM) with coincubating with SJP or LY294002 or the both in prior. The images are presented with original magnification 200×. CG: control group, OG: oleic acid group, and SG: SJP treatment group. (b) Western blot of Akt/pAkt (Ser473, Thr308), Mtor/pmTOR, S6/pS6, SREBP1-c, and FASN. CG: control group, OG: oleic acid group, SG: SJP treatment group, IG: inhibitor (LY294002) group, SIG: SJP+ LY294002 group. The left column exhibited the western blotting bands; the right column showed the relative expression of target genes in experimental groups. *∗*: there is a statistical significance between groups (p<0.05).

Oleic acid (0.5mM) stimulated LO2 cells were employed to mimic a cell model of NAFLD. Abundant of lipid droplets were observed in LO2 cells by oil red O staining after coincubating with oleic acid for 24h, and the lipid accumulation was significantly ameliorated by SJP treatment. (Figure 3(a)) Although 0.5mM oleic acid stimulation had no influence to cell viability, high expression of pAkt (Ser473), pmTOR, pS6, and FASN was induced, which was restored by SJP treatment. Inhibition of PI3K by LY294002 inhibited oleic acid induced Akt activation, but the activation of mTOR and S6 was not altered in this oleic acid induced NAFLD cell model. In addition, these data suggested the activation of mTOR pathway is not via PI3K/Akt pathway (Figure 3(b)).

## 5. Discussion

In the present study, we successfully induced NAFLD by high fat diet feeding. Plenty of lipid droplets and distinct damages were observed in the ultrastructure of liver cells. Lower level of tissue antioxidants and more severe pathological injuries were detected as well. However, SJP treatment significantly attenuated the ultrastructure changes and protected rat liver against inflammatory injury caused by HFD feeding induced hepatic steatosis. Additionally, SJP attenuated lipid accumulation in oleic acid stimulated LO2 cells via inhibiting the activation of Akt/mTOR/S6 pathway.

NAFLD, a disease often caused by excessive intake of high fat and calories, is supposed to happen following a clear pathway according the theory of traditional Chinese medicine [21]. And that excessive nutrients intake causes damage to the transport function of spleen which is responsible for normal transportation and spreads of nutrients depending on normal motion of spleen qi. Then, spleen qi stagnation further causes phlegm retention and blood stasis and finally blocks the liver collaterals and induces the development of NAFLD [30]. SJP, composed by Jiangchan (*Bombyx Batryticatus*), Chantui (*periostracum cicada*), Jianghuang (*Curcuma longa*), and Dahuang (raw rhubarb), first derived from the famous Chinese medical book “wan bing hui chun” and finally recorded in “shang han wen yi tiao bian”, which was compiled by Li-shan Yang during the Qing dynasty of China, was applied as a common therapeutic Chinese herbal medicine formula in the treatment of syndrome “depression of sanjiao fire and blocked motion of qi”, especially liver qi stagnation as all of the four components of SJP belong to the liver meridian according to the pharmacopoeia [22]. The whole formula is effective in harmonizing liver and spleen and regulating qi-activity, which was supposed to be the therapeutic principle of NAFLD according to the pathogenesis of traditional Chinese medicine. Therefore, SJP was empirically employed to treat NAFLD in China, and it showed significant efficacy in ameliorating hepatic enzyme increase, insulin resistance, and metabolic disorder as well as lowering body weight from the results of present research [24, 25, 31]. Here in the present study, we established a rat model of NAFLD (to be exactly, we established a simple liver steatosis rat model as there was no change in serum hepatic enzymes and we found unconspicuous inflammatory cell infiltration in pathological images as well) through HFD feeding. Obvious changes were observed in both appearance and ultrastructure of liver with characters of liver steatosis and SJP was able to prevent or attenuate these changes just as expected.

The exact pathogenesis of NAFLD is still unknown, but accumulating evidence has indicated important roles of oxidative stress, mitochondrial dysfunction, insulin resistance, endoplasmic reticulum stress, and chronic inflammation [32–35] and these factors always interact with each other and finally lead to the occurrence and development of NAFLD. Oxidative stress is often initiated by abundant production of ROS and is considered an important contributor to hepatocyte injury associated with NAFLD [36]. There is a significant increase in ROS when NAFLD exists and this may be related to mitochondrial dysfunction as mitochondrial respiratory chain is the main subcellular source of ROS [33, 37]. Additionally, endoplasmic reticulum stress is another contributor to increased ROS [38]. Excessive ROS leads to the production of lipid peroxides such as MDA, which may aggravate oxidative stress and mitochondrial injury. In the present study, HFD feeding causes both obesity and liver steatosis, which were considered to be tightly related in prevalence and pathogenesis. Known to all, obesity is a status with chronic, low-grade inflammation. Lipid over accumulation and energy metabolism disorder lead to overexpression of proinflammatory cytokines and subsequent activation of inflammatory signal pathway which promote the oxidative stress process and finally lead to liver damage [39]. Similarly, as excessive lipid accumulation in hepatocyte, a large number of free fatty acids oxidated in mitochondrial and then a large amount of ROS produced [34]. Excessive ROS cause damage to mitochondrial and mitochondrial dysfunction lead to subsequent production of ROS and lipid peroxide [40]. Although we did not find apparent increase in ROS and MDA in the present study, we did find obvious decrease in SOD and GSH-px, which are important members in antioxidant system capable of protecting cell membrane against oxidative damage. From our point of view, lipid accumulation in hepatocyte caused damage to the mitochondrial and led to subsequent production of ROS and lipid peroxide and at the same time the antioxidant system might be initiated to neutralize ROS and other lipid peroxides. As a permanent consumption, an obvious decrease of SOD and GSH-px was observed instead of a significant increase of ROS and MDA. And this may also interpret unchanged serum hepatic enzymes and nontypical inflammatory cell infiltration in liver tissue we observed. However, we did find some changes in the ultrastructure of liver cells such as endoplasmic reticulum expansion, mitochondrial contraction, and cell matrix solidification. Fortunately, SJP in the present study showed great efficacy in lowering body weight, reducing intracellular lipid droplets, increasing tissue antioxidants level, attenuating hepatocyte swelling and vacuolation, and protecting subcellular structure injuries, just as the previous studies have demonstrated.

Current research believed that insulin resistance played an important role in the pathogenesis of NAFLD [8]. PI3K/ Akt signaling pathway is one of the main downstream pathways of insulin and Akt is the key signaling transduction molecule in PI3K pathway. Physically, insulin induces the upstream activation and then Akt phosphorylation which further mediates glycogen synthesis, glycolysis, glucose transporter, protein synthesis, and lipid synthesis. Also, some researches demonstrated that Akt could directly inhibit the gene expression of fatty acid oxidation and thus regulate liver lipid metabolism [41]. Therefore, PI3K/Akt/mTOR signaling pathway has garnered much interest in NAFLD research but present results have not reached a consistent view on the role it played on NAFLD. Although many evidence indicates that activation of phosphatidylinositol 3-kinase (PI3K)/AKT pathway is associated with marked accumulation of intracellular lipid droplets and promotion from NASH to fibrosis [42, 43], some studies revealed that PI3K/Akt activation is benefit for ameliorating insulin resistance [44], oxidative stress [45], and lipid accumulation [46]. In our study, we found that HFD induced NAFLD was related to high expression of pAkt (Ser473), pmTOR, and pS6, while the activation of mTOR/S6 pathway was not via PI3K/Akt pathway. And we believe that there might be some other pathways involved in the activation of Akt besides insulin signal pathway as we detected no difference in serum insulin concentration between rats with NAFLD and the controls. Additionally, studies also demonstrated that some herbs or herbal extracts ameliorated NAFLD through regulating PI3K/Akt pathway [9], which were in accordance with our results that SJP could reduce Akt phosphorylation and decrease lipogenesis in liver cells. However, Scutellarin was found to protect against NAFLD through PI3K/Akt and related nuclear factor Nrf2 activation [4]. Thus, the specific role of Akt in NAFLD still needs further investigation.

In the present study, 12-weeks HFD feeding only caused simple steatosis without liver inflammation and insulin resistance. The effect of SJP we obtained here was limited in simple steatosis. Prolonged feeding or MCD diet feeding might help explore the effect of SJP on more severe NAFLD such as NASH or fibrosis.

In conclusion, SJP is efficient in attenuating HFD induced NAFLD in rats and this effect might be partly related to the inhibition of Akt/mTOR/S6 pathway.

## Figures and Tables

**Table 1:** Expression of tissue inflammatory agents and serum hepatic enzymes in the three experimental groups.

	**NG (n=6)**	**HFG (n=8)**	**SG (n=8)**
ALT(U/L)	65±36	67±30	65±17
AST(U/L)	270±89	248±86	285±76
FBG (mmol/L)	4.4±0.5	4.5±0.5	5.1±0.8
INS (*μ*IU/ml)	95±6	85±15	96±17
Pathological score	14.3±7.1	45.6±12.8^*∗*^	20.1±18.6^#^

ALT: alanine aminotransferase; AST: aspartate aminotransferase; FBG: fasting blood glucose; INS: insulin. ^*∗*^: compared with NG, p<0.05; ^#^: compared with HFG, p<0.05.

## Data Availability

The data used to support the findings of this study are included within the article.
